# Cold Dialysis Combined with Intradialytic Exercise Improves Insulin Sensitivity in Hemodialysis Patients: A Pilot Randomized Clinical Trial

**DOI:** 10.3390/medicina62071268

**Published:** 2026-06-30

**Authors:** Argyro A. Krase, Christoforos D. Giannaki, Ilias Ntoumas, Ioannis Stefanidis, Christina Karatzaferi, Giorgos K. Sakkas

**Affiliations:** 1 Department of Physical Education and Sport Science, School of Physical Education, Sports and Dietetics, University of Thessaly, 42100 Trikala, Greece; arg.krase@gmail.com (A.A.K.); intoumas@uth.gr (I.N.); ck@uth.gr (C.K.); 2 Department of Life Sciences, School of Life and Health Sciences, University of Nicosia, Nicosia 2417, Cyprus; giannaki.c@unic.ac.cy; 3 Division of Nephrology, Department of Medicine, School of Health Sciences, University of Thessaly, 41110 Larissa, Greece; stefanid@med.uth.gr

**Keywords:** cold dialysis, hemodialysis, intradialytic exercise, insulin sensitivity, glucose metabolism, randomized clinical trial, chronic kidney disease

## Abstract

*Background and Objectives*: Hemodialysis patients are characterized by insulin resistance. Cold dialysis (CD), with or without intradialytic exercise training (IET), can be beneficial for overall health in this population. The present study aimed to examine the long-term effect of the combined effect of cold dialysis and intradialytic exercise training on glucose disposal and various health and quality-of-life parameters in hemodialysis patients. *Materials and Methods*: Fourteen stable hemodialysis patients were randomly allocated in one of the two groups: the Cold Dialysis (35 °C) + Exercise Group (N = 7) (CD) and the Standard dialysis (37 °C) + Exercise Group (N = 7) (SD). Glucose disposal and insulin sensitivity, body composition, functional capacity, and quality of life were assessed before and after a 7-month intervention period. *Results*: Insulin sensitivity was improved by 16.7% in the CD group compared to the SD group. The area under the curve (AUC) for glucose and insulin disposal rate was also improved in the CD group compared to the SD group. Functional capacity and quality-of-life indices improved significantly in both groups (all *p* ≤ 0.05) independent of dialysate temperature. *Conclusions*: Seven months of cold dialysis combined with intradialytic exercise training was associated with improvements in insulin sensitivity and glucose metabolism in stable hemodialysis patients. Long-term exercise training has proven benefits for hemodialysis patients’ health that can be further enhanced by cold dialysis. *Trial Registration:* The study is registered at ClinicalTrials.gov (NCT03905551).

## 1. Introduction

Abnormal glycemic control is a common feature in hemodialysis patients that is often associated with high morbidity and mortality [1]. Insulin Resistance is characterized by resistance to the effects of insulin on glucose uptake, metabolism, or storage. This effect is manifested by decreased insulin-stimulated glucose transport and metabolism in adipocytes and skeletal muscle and by impaired suppression of hepatic glucose output [2]. Many factors have been implicated in the pathogenesis of increased IR, including anemia, physical inactivity, uremic myopathy, and numerous potential uremic toxins, particularly of the middle molecule variety, with these factors leading to the reduction in quality of life in hemodialysis patients [3].

Chronic kidney disease and hemodialysis represent major global public health challenges associated with increased cardiovascular risk, reduced functional capacity, impaired quality of life, and substantial healthcare burden. Modern hemodialysis care requires a multidimensional and multidisciplinary clinical approach focused not only on dialysis adequacy, but also on metabolic health, physical functioning, symptom management, and patient-centered outcomes [4].

Exercise can serve as an effective and safe method for improving various health parameters in hemodialysis patients [5]. Benefits include enhancements in parameters related to the cardiovascular system, increase/maintenance of skeletal muscle mass, increased muscle strength and exercise capacity, better regulation of blood pressure, improved bone mineral metabolism, improved functional capacity, better sleep quality, and enhanced psychological parameters including reduced anxiety and depression [5,6,7]. These beneficial adaptations and improvements can frequently lead to an enhanced quality of life [8], reduced fatigue [9], and reduced hospitalization [10], as well as a decrease in the high mortality rates prevalent in this population [11]. Additionally, exercise can lead to a series of adaptations that improve insulin sensitivity and reduce insulin resistance [9]. This is particularly important in populations such as the hemodialysis patients characterized by high rates of type II diabetes and insulin resistance, conditions that have significantly negative consequences on their cardiovascular health and already low quality of life [3].

Lowering the dialysate temperature (i.e., 35–36 °C), also called cold dialysis (CD) has been proposed as a simple and useful method to improve many aspects of health in patients undergoing hemodialysis [12,13]. The CD has been employed as a countermeasure to reduce intradialytic hypotension episodes by increasing peripheral resistance, leading to increased intradialytic mean arterial pressure without jeopardizing dialysis adequacy [14]. In addition, there is accumulated evidence to suggest that CD can also reduce overall cardiovascular mortality [14,15]. In general, hemodialysis patients tolerate long-term CD very well, reporting high levels of satisfaction (approximately 80%), less fatigue, faster recovery times after dialysis, feeling more energetic with better cognitive capacity, and having the overall sensation that their general health has significantly improved [16].

Previous findings demonstrated that combining an exercise program during haemodialysis with cold dialysis (35 °C) can acutely improve glucose disposal and insulin sensitivity [17,18]. However, the long-term effects of this non-pharmacological intervention on insulin- and glucose-related markers, as well as on health and quality-of-life indicators in this specific population, are not yet known.

Accordingly, the primary objective of this pilot randomized clinical trial was to evaluate whether a 7-month intervention combining cold dialysis with supervised intradialytic exercise training could improve insulin sensitivity, glucose metabolism, physical functioning, and health-related quality of life among clinically stable patients undergoing maintenance hemodialysis.

## 2. Materials and Methods

### 2.1. Trial Design

The present study was conducted as a pilot randomized clinical trial and is reported in accordance with the CONSORT 2010 Statement and its accompanying checklist for randomized trials.

### 2.2. Study Population

**Figure 1:**
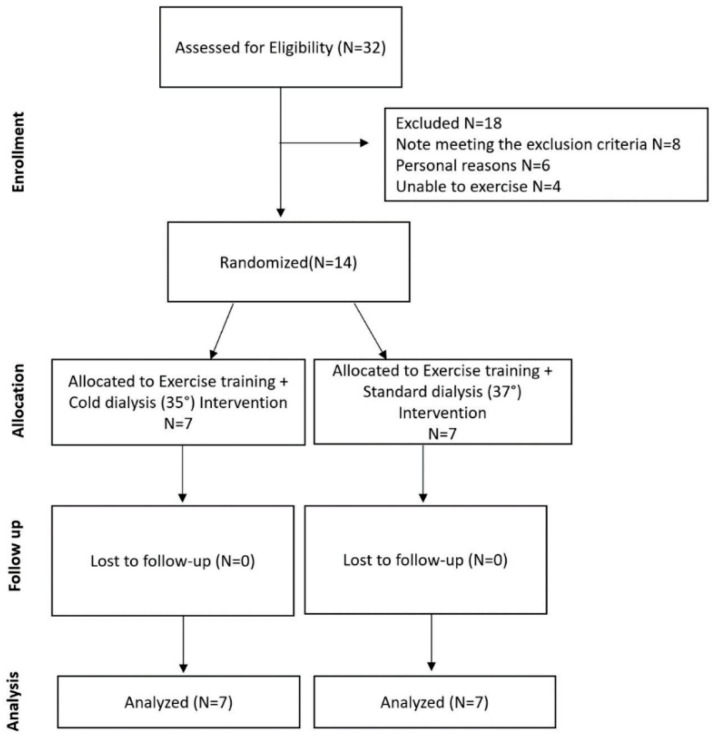
CONSORT flow diagram presenting participant screening, enrollment, randomization, allocation, follow-up, and final analysis of the study population.

Fourteen hemodialysis patients voluntarily participated in this study (Figure 1). Inclusion criteria included clinically stable condition, receiving regular dialysis treatment for at least 3 months, with adequate dialysis delivery Kt/V > 1.2 and good compliance of dialysis treatment, serum albumin > 2.5 g/dL, hemoglobin ≥ 11 g/dL, and treatment with erythropoietin. Exclusion criteria included reason to be in a catabolic state, such as hyperthyroidism, active vasculitis, malignancies, pregnant, HIV, opportunistic infections, musculoskeletal contraindication to exercise, requirement for systemic anticoagulation, participant or participated in an investigational drug or medical device study within 30 days or five half-lives or inflammations, that required intravenous antibiotics within 3 months prior to enrollment, diabetics receiving insulin therapy, New York Heart Association grade IV heart failure, and mental incapacity to consent.

The study was approved by the Human Research and Ethics Committee of the University of Thessaly (GR/921-4/11 May 2014), and by the bioethics committee of the individual dialysis centers. After being thoroughly informed about the procedures, all patients gave their written informed consent prior to study participation.

### 2.3. Study Design

Patients were recruited from two hemodialysis units from the area of Central Greece. The study was performed from February 2015 to February 2020. All participants received 7 months of supervised intradialytic aerobic exercise training by a clinical exercise physiologist. The primary intervention difference between groups was the dialysate temperature during hemodialysis sessions, while both groups followed the same supervised intradialytic exercise training protocol and standard clinical dialysis care. Patients were randomized using a computer-generated randomization procedure and assigned to either the Cold Dialysis plus Intradialytic Exercise group or the Standard Dialysis plus Intradialytic Exercise group: the Cold Dialysis (35 °C) + Intradialytic Exercise Group (N = 7) (CD) and the Standard dialysis (37 °C) + Intradialytic Exercise Group (N = 7) (SD). All patients were assessed for their body composition, functional capacity, blood pressure, and insulin sensitivity. Quality of life and fatigue severity were assessed by validated questionnaires. All measurements were performed after the completion of the hemodialysis session and before and after the 7 months experimental intervention. The primary outcomes of the study were changes in insulin sensitivity and glucose homeostasis indices, including OGIS and glucose/insulin AUC responses. Secondary outcomes included body composition, functional capacity, blood pressure, fatigue severity, and quality-of-life parameters.

### 2.4. Intradialytic Exercise Training

During the intradialytic exercise training, patients performed cycling for 60 min in the supine position. During the sessions, the patients were asked to pedal at 45 rpm for the first 10 min and then to 60 rpm on a bedside cycle ergometer (Model 881 Monark Rehab Trainer, Monark Exercise AB, Varberg, Sweden). Patients cycled at approximately 60% of their pre-assessed maximum power capacity. The exercise regime started 1 h after the beginning of the hemodialysis session. The patients’ maximum power capacity was determined by a modified version of the Åstrand Bicycle Ergometer test protocol at bed-side on a previous dialysis session. Exercise was well tolerated by all patients, and no adverse reactions were reported, apart from some irregular muscle cramps.

### 2.5. Body Composition Assessment

The patient’s whole body and regional fat and lean body mass were measured by Body Composition Monitor (BCM—Fresenius Medical Care Deutschland GmbH, Bad Homburg vor der Höhe, Germany), which has been specifically designed for patients with kidney failure [19].

### 2.6. Functional Capacity Assessment

To assess the functional capacity of patients, several tests commonly used in studies with exercise for hemodialysis patients were utilized. Specifically, two sit-to-stand tests (STS-5 and STS-60), the 6-min walk test (6MWT), a 6-m walking test, and the handgrip test were employed. For the STS-5 test, participants were instructed to complete five consecutive sit-to-stand movements from a chair as rapidly as possible, with the total time required (in seconds) recorded as the performance outcome. The STS-60 test requires the patient to stand up and sit down as quickly as possible from a chair for 60 s, with repetitions counted. During the 6MWT, the patient walks for 6 min, and the distance covered is measured in meters. Additionally, the time taken to walk a distance of 6 m at a fast pace (fast walk) was measured as an indicator of everyday functional capacity. Finally, maximal isometric handgrip strength, reflecting the strength of the hand and forearm muscles, was evaluated using an electronic hand dynamometer. In this test, patients hold the dynamometer in the hand to be tested, with their arms hanging by their sides. When ready, they squeeze the dynamometer with maximum isometric effort, maintaining it for about 5 s. Strength in this test is measured in kilograms [20].

### 2.7. Questionnaires

Assessment of health-related quality of life was performed using the 36-Item Short Form Health Survey (SF-36), a questionnaire previously validated in patients undergoing dialysis [21]. Fatigue severity was assessed by using Fatigue Severity Scale [22]. All questionnaires were administrated and supervised by experienced personnel using the interview method.

### 2.8. Insulin Sensitivity Assessment

Glucose tolerance was assessed using a 75 g, 2 h oral glucose tolerance test following an overnight fast by measuring the area under the glucose-and-insulin curve (AUC) using a trapezoidal integration [23]. Venous insulin and glucose blood samples were collected at 0, 30, 60, 90, and 120-min following ingestion of the 75 g of glucose dissolved in 400 mL of water. The Oral Glucose Insulin Sensitivity (OGIS) index was used to estimate insulin sensitivity [24]. All metabolic assessments were performed under standardized testing conditions before and after the 7-month intervention period and following completion of the hemodialysis session in both groups. Metabolic testing was performed separately from the intradialytic exercise sessions [25,26].

### 2.9. Basal Measurements

Blood pressure, heart rate (Polar V800, H7, Polar Electro Oy, Kempele, Finland), and exhaling air nitric oxide (NO—Quark NObreath, COSMED, P/N:C09082-01-99, Rome, Italy) were assessed before, at each hour, and after hemodialysis session in both groups.

### 2.10. Biochemical Analysis

Albumin, hemoglobin (Hb), and hematocrit (Hct) blood analyses were performed at the hemodialysis clinical laboratory of the General Hospital of Trikala under standard hospital procedures.

### 2.11. Statistical Analysis

The statistical analysis was performed using IBM SPSS Statistics version 29.0 (IBM Corporation, Armonk NY, USA). A Multivariate Analysis of Variance (MANOVA) followed by post-hoc paired-samples t tests were used to assess the effects of time (pre/post) and protocol. MANOVA was used as an exploratory approach to examine overall time and group effects across related outcome variables. The MANOVA results demonstrated no statistically significant main or interaction effects. Nevertheless, given the exploratory design and limited sample size of this pilot trial, post-hoc paired comparisons and effect size analyses were additionally conducted to further investigate potential within-group changes and clinically meaningful adaptations. To further strengthen the analysis, Cohen’s d effect sizes (0.2–0.5: small effect; 0.5–0.8: moderate effect; >0.80: large effect) were also used to identify paired differences between protocols and times. Δ-change differences were assessed by a General Linear Model (GLM)-Multivariate test. To assess normality, the Shapiro-Wilk test was used alongside graphical representations, including the Normal Q-Q plot, Detrended Normal Q-Q plot, and Box Plot. The significance level was set at 5%. Beyond significance testing (*p*-value), effect size was also considered to evaluate the magnitude of the effect. Due to the exploratory and pilot nature of the present randomized clinical trial, emphasis was also placed on effect size interpretation alongside statistical significance. All statistical analyses were conducted using a per-protocol approach, since all participants completed the intervention and follow-up assessments. Given the exploratory pilot nature of the study and the multiple outcomes assessed, the findings should be interpreted cautiously due to the increased risk of type I error.

### 2.12. Power Analysis

A priori sample size calculations were conducted based on plasma glucose at 120 min, mg/dL in the hemodialysis patients from a previous published article [26]. The resulting minimum required sample size was six for two-sided type 1 and type 2 errors at 5%. Sample size calculations were conducted using G*Power 3.1 [27].

### 2.13. Trial Registration

The trial was registered under the title “Impact of Cold Dialysis in Combination with Intradialytic Exercise in Aspects Related to Quality of Life and Health (REACD)” with the full protocol accessible through ClinicalTrials.gov website. Trial registration number: NCT03905551. The trial was registered at ClinicalTrials.gov under the title “Impact of Cold Dialysis in Combination with Intradialytic Exercise in Aspects Related to Quality of Life and Health (REACD)” (Identifier: NCT03905551). The trial was registered retrospectively after participant enrollment had commenced due to an administrative oversight during the study initiation process. At the time the study was initiated, institutional procedures primarily focused on obtaining Ethics Committee approval before participant recruitment, which had been completed before enrollment began. Prospective public trial registration had not yet been fully incorporated into the local administrative workflow for this type of clinical intervention study. Importantly, no changes were made to the study protocol, eligibility criteria, intervention procedures, outcome measures, or statistical analysis plan following trial registration.

## 3. Results

Characteristics of the study population are presented in Table 1. Briefly, 14 stable hemodialysis patients (10 men/4 women), fulfilled the eligibility criteria and participated in the study (Figure 1). There were no statistically significant differences observe between groups before and after the 7 months of intervention on Haematocrit, Haemoglobin, and body composition parameters (*p* > 0.05). Figure 1 presents the CONSORT flow diagram summarizing participant enrollment, randomization, follow-up, and inclusion in the final analysis.

**Figure 2:**
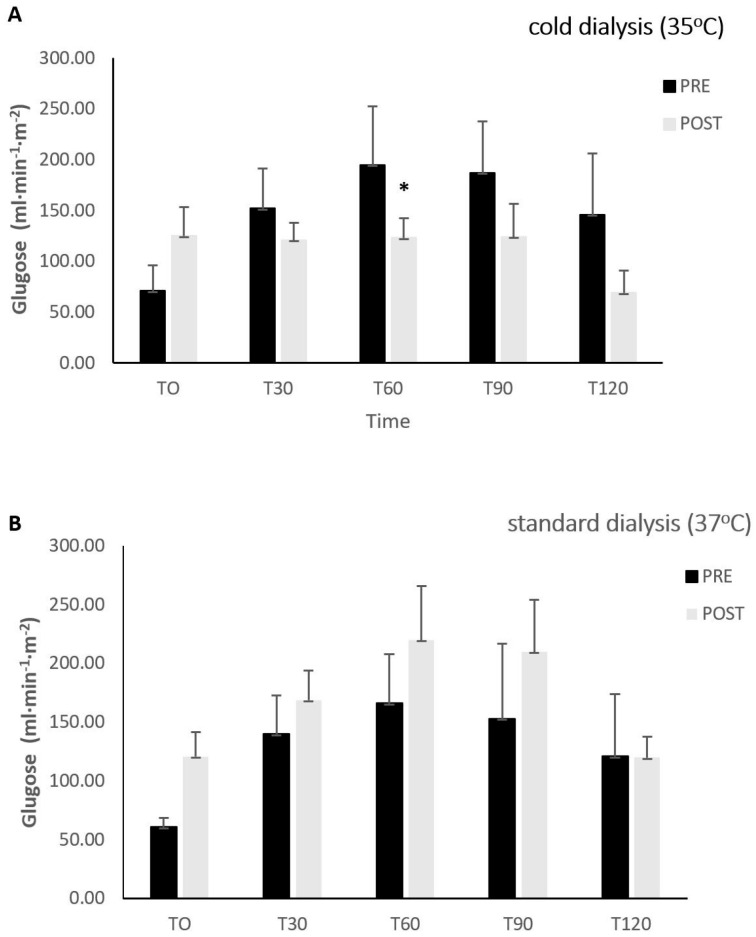
Oral glucose tolerance test responses at baseline and after the 7-month intervention in the cold dialysis (CD) and standard dialysis (SD) groups. Blood glucose concentrations were assessed at 0, 30, 60, 90, and 120 min following ingestion of 75 g of glucose dissolved in 400 mL of water. Data are presented as mean ± SD. * Statistically significant difference observed between groups at 60 min post intervention (*p* = 0.025). Data are presented as mean ± SD. (**A**) Cold dialysis (35 °C) group; (**B**) Standard dialysis (37 °C) group.

**Figure 3:**
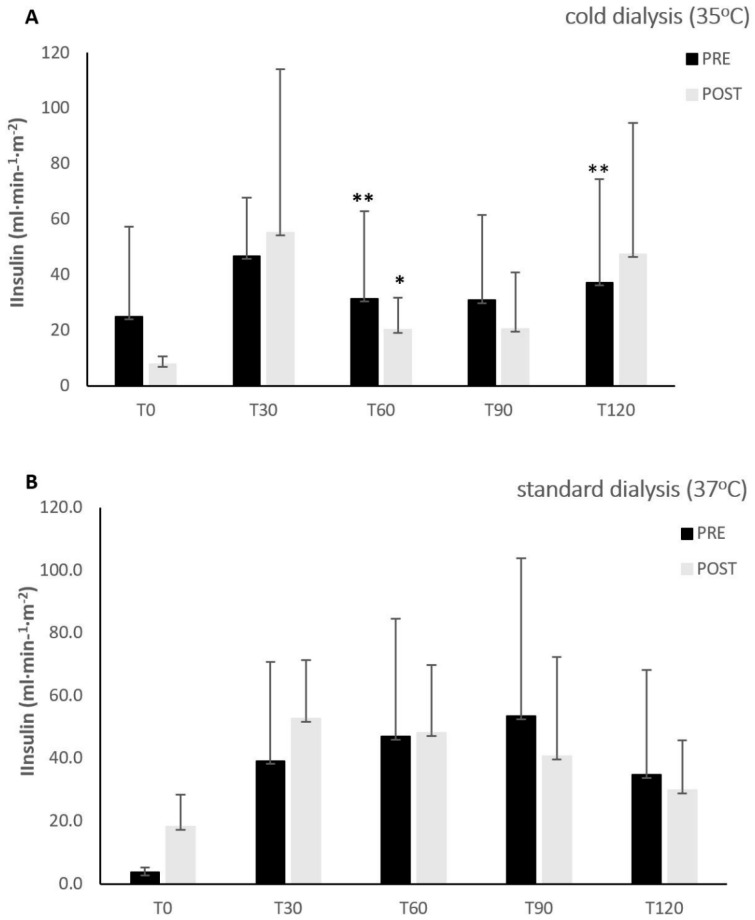
(**A**,**B**) Insulin responses during the oral glucose tolerance test at baseline and after the 7-month intervention in the cold dialysis (CD) and standard dialysis (SD) groups. Insulin concentrations were measured at 0, 30, 60, 90, and 120 min following ingestion of 75 g of glucose dissolved in 400 mL of water. Data are presented as mean ± SD. ** Statistically significant difference observed between groups at 60 min post-intervention (*p* = 0.013). * Differences between groups at baseline were observed at the 60 min (*p* = 0.051) and 120 min (*p* = 0.031) time points. Data are reported as mean ± SD.

Our findings showed that the rate of glucose and insulin disposal during oral glucose tolerance test was improved in the CD group compared to the SD group after 7 months of intervention (*p* < 0.050, d > 0.80) (Table 2, Figure 2 and Figure 3). More specifically, the OGIS was improved by 16.77% in the CD group compared to the SD group. The rate of insulin and glucose disposal as measured by method of AUC improved significantly. A lower AUC_Insulin_ rate suggests better tissue responsiveness to control blood glucose, especially in muscle and fat.

Also, AUCglucose improved by −35.59% following the CD intervention compared with SD (Figure 2 and Figure 3). In addition, post-dialysis systolic blood pressure improved statistically significantly in the CD group (*p* = 0.05) compared to the SD group (Table 3).

Functional capacity improved in both groups after 7 months of intervention, independently of dialysate temperature (Table 4). Specifically, STS-60, as well as the distance in the 6MWT, was increased significantly in both groups (*p* = 0.050; SD: *p* = 0.020). Performance in STS5, 6 m fast walking test, and hand grip were found to be slightly improved in both groups after the intervention. However, this improvement did not reach the statistically significant level (Table 4).

Quality of life improved in both groups, while fatigue score did not reach the statistically significant level (Table 5).

## 4. Discussion

The present study sought to assess for the first time the chronic effects of CD combined with intradialytic exercise training in parameters related to insulin sensitivity and exercise capacity of hemodialysis patients. The present results suggest that cold dialysis combined with supervised intradialytic exercise training may contribute to improvements in insulin sensitivity and metabolic regulation in stable hemodialysis patients. As anticipated, exercise training improved overall exercise capacity and quality-of-life indices in both groups, irrespective of dialysate temperature. Importantly, because both groups participated in supervised intradialytic exercise training, the independent effects of cold dialysis cannot be fully isolated, and exercise likely contributed substantially to the observed metabolic and functional adaptations. Nevertheless, these findings should be interpreted with appropriate caution, as the limited sample size and the numerical baseline differences between the study groups, particularly with respect to age and baseline OGIS values, may have influenced the observed metabolic responses despite the randomized study design. It is well established that glucose intolerance in hemodialysis patients is associated with insulin resistance in peripheral tissues, such as skeletal muscle and adipose tissue [2,2]. In addition, previous studies have indicated that hemodialysis per se can improve carbohydrate metabolism in chronic uremia by ameliorating glucose intolerance associated with increased tissue sensitivity to insulin [28]. Τhe mechanism of improving the glucose tolerance and insulin resistance of uremic patients after the initiation of the hemodialysis treatment has not been well established; however, it seems that chronic uremic toxicity enhances insulin resistance, which eventually leads to diabetes [2,3,26].

Cooling the dialysate temperature improves hemodynamic tolerability of hemodialysis and exerts a protective effect over major organs [12,16]. Previous work from our group demonstrated that cold dialysis improved thermoregulatory responses and reduced hypovolemic stress during hemodialysis [29]. If glucose uptake is decreased as a response to cold hemodialysis, it could be a transient adjustment in the body’s metabolic activity due to the cooling effect, as the body reduces overall energy expenditure. In theory, this might help prevent excessive glucose consumption, especially in patients with fluctuating glucose levels, like those with diabetes [30]. However, the long-term effect reduction of glucose uptake in tissues like muscle and fat can lead to hyperglycemia, which is undesirable. If the body is unable to effectively take up and utilize glucose, it can worsen insulin resistance, a common issue in patients with kidney disease and diabetes [31]. The effects of cold hemodialysis on glucose metabolism are still not fully understood, and there is no strong evidence to suggest that decreased glucose uptake is a beneficial long-term adaptation [12].

Recent findings document that cold exposure activates brown adipose tissue via adrenergic stimulation, which combusts significant amounts of blood glucose and free fatty acid to produce heat [32]. Many studies suggest that brown adipose tissue activation improves insulin sensitivity [33]. The investigators’ central hypothesis is that cold-induced brown adipose tissue activation increases whole-body insulin sensitivity in humans via augmented plasma glucose and free fatty acid clearance [33]. Also, stimulation of energy expenditure by cold exposure or exercise training generally leads to decreased plasma insulin levels and an improvement in glucose tolerance, suggesting that insulin action on peripheral tissues increases when energy expenditure is stimulated [34]. Also, the improvement of muscle training under cooling conditions might be due to the fraction of cardiac output perfusing in cutaneous circulation that reduced in presence of cooling [35].

Based on the above, we hypothesized that cooling the dialysate temperature could improve insulin sensitivity in hemodialysis patients. Indeed, our results showed a 16.77% improvement in OGIS and 35.5% in glucose metabolism in the CD group compared with the SD group potentially through metabolic adaptations that require further investigation, and secondarily by increasing the responsiveness of peripheral tissues to insulin [34]. On the other hand, a colder dialysate solution may induce an activation to the sympathetic nervous system and brown adipose tissue metabolism, resulting in an improvement in glucose metabolism without affecting pancreatic insulin secretion [32]. However, these proposed physiological mechanisms should be interpreted cautiously, since no direct mechanistic measurements or biomarker assessments were performed in the present study. Many studies have demonstrated beneficial effects of cold dialysis on hemodynamic stability and overall patient health [14]. Lower dialysate temperatures may influence peripheral circulation, sympathetic nervous system activity, and metabolic regulation, potentially affecting insulin sensitivity and glucose metabolism. However, the exact physiological mechanisms underlying the observed metabolic adaptations remain incompletely understood and were not directly assessed in the present study [12].

Other physiological adaptations of CD include reduced heart rate, cardiac output, and stroke volume, leading to high arterial blood pressure and thus a greater total peripheral resistance after hemodialysis [12,36]. For instance, Mustafa et al. (2016) showed that the rate of intradialytic hypotension was reduced by 70% with CD compared with SD [36]. Moreover, the reduction of arterial blood pressure at the end of the hemodialysis session is another aspect of an independent risk factor for cardiovascular mortality in these patients and significantly affects their post-dialysis energy levels and overall quality of life. Our results showed clinical improvements in systolic blood pressure in post-dialysis hours in the CD group compared to the SD group.

Also, the available evidence suggests no negative effect on dialysis adequacy, with an increase in symptoms of discomfort of unclear severity during CD [37]. In general, patients receiving CD reported high levels of satisfaction (approximately 80%) [16], less fatigue, faster recovery times after hemodialysis, feeling more energetic with better cognitive capacity, and having the overall sensation that their general health has dramatically improved [38].

The present results confirm previous studies that examined the effect of chronic intradialytic exercise training on health, functional capacity, and quality of life [6,9,39]. Both groups presented a significant improvement in functional capacity and quality of life, but no differences were observed in body composition parameters. It is widely recognized that exercise confers multiple benefits for patients undergoing hemodialysis by improving various physiological systems, thereby enhancing physical functioning, muscular strength, and endurance. Moreover, exercise has positive effects on psychological, cognitive, and mental parameters, ultimately contributing to an improved health-related quality of life [5,9].

### 4.1. Perspectives for Practice

The present findings may have practical implications for multidisciplinary hemodialysis care aiming to improve metabolic health and patient-centered outcomes. Non-pharmacological strategies such as intradialytic exercise and individualized dialysis modifications may represent feasible supportive approaches for improving functional capacity, glucose regulation, and quality of life in stable hemodialysis patients. However, larger studies are required before routine clinical implementation can be recommended [40]. From a clinical perspective, these findings should be interpreted within the broader framework of multidisciplinary hemodialysis care, where metabolic risk, cardiovascular stability, physical functioning, fatigue, and patient-reported quality of life are closely interrelated [4]. Current CKD guidance emphasizes comprehensive risk reduction and encourages physical activity according to cardiovascular tolerance and frailty status, supporting the integration of exercise-based strategies into nephrology care [41]. Recent evidence also suggests that intradialytic exercise can improve physical and psychological outcomes in hemodialysis patients, whereas lower dialysate temperature may reduce intradialytic hypotension and improve hemodynamic stability [36], although discomfort and patient tolerability remain important considerations [42]. Therefore, the present findings should be considered preliminary but clinically relevant, suggesting that combining individualized dialysate temperature strategies with supervised intradialytic exercise may represent a feasible adjunctive approach for selected stable hemodialysis patients. Larger, adequately powered randomized trials are required before these strategies can be recommended as routine clinical practice.

### 4.2. Strengths and Limitation

The present study has several strengths, including the relatively long intervention period, the supervised implementation of intradialytic exercise training by specialized exercise professionals, and the randomized clinical trial design. In addition, there is currently limited evidence regarding the long-term effects of cold dialysis combined with intradialytic exercise on metabolic regulation and insulin sensitivity in hemodialysis patients.

Although this study provides encouraging preliminary findings, several limitations should be considered when interpreting the results. The relatively modest sample size inevitably reduced the statistical power of the analyses and limited the generalizability of the findings, despite the randomized study design and the moderate-to-large effect sizes observed across several outcome measures. In addition, although patients were randomized, the small sample size may have contributed to baseline numerical differences between groups, particularly in age and baseline metabolic parameters.

Furthermore, mechanistic pathways related to thermoregulation, sympathetic nervous system activation, brown adipose tissue activity, and inflammatory responses were not directly assessed. The absence of inflammatory and metabolic biomarkers, including HbA1c and inflammatory cytokines, limits the ability to fully interpret the physiological mechanisms underlying the observed metabolic adaptations. Another limitation is the increased possibility of type I statistical error due to the multiple comparisons performed in the present exploratory pilot trial. Although additional statistical approaches such as ANCOVA or mixed-model analyses adjusted for baseline values could provide further insight, these analyses were not applied due to the small sample size and the potential risk of unstable statistical estimates. Accordingly, the present findings should be interpreted with caution and regarded as preliminary, serving primarily to generate hypotheses for future research.

### 4.3. Future Directions

Future adequately powered multicenter randomized clinical trials are required to confirm the present findings and further investigate the physiological and clinical effects of combining individualized dialysate temperature strategies with supervised intradialytic exercise training in hemodialysis patients. Future studies should also include mechanistic assessments related to thermoregulation, brown adipose tissue activation, autonomic nervous system regulation, inflammatory biomarkers, and long-term cardiovascular outcomes in order to better clarify the clinical relevance of cold dialysis interventions in nephrology care.

## 5. Conclusions

This pilot randomized clinical trial suggests that cold dialysis combined with supervised intradialytic exercise training may improve insulin sensitivity and glucose regulation in stable hemodialysis patients. In addition, supervised intradialytic exercise was associated with improvements in functional capacity and health-related quality of life. Although the present findings are preliminary and limited by the small sample size, the combined intervention appears feasible and clinically promising as a supportive non-pharmacological strategy in hemodialysis care. Future adequately powered randomized clinical trials with larger study populations are warranted to validate the present findings and further elucidate the physiological mechanisms underlying the observed effects.

## Figures and Tables

**Table 1:** Patient’s characteristics according to study allocation.

Variables	CD		SD	
	PRE (First Training)	POST (Pre Last Training)	PRE (First Training)	POST (Pre Last Training)
N	7		7	
Male/Female	3/4		3/4	
Years in hemodialysis	8.14 ± 2.90		8.71 ± 3.10	
Height (cm)	172.8 ± 7.40		167.1 ± 5.76	
Age (yr)	63.57 ± 9.93		52.71 ± 16.84	
Hct	35.58 ± 3.02		35.94 ± 2.91	
Hb (g/dL)	11.14 ± 1.14		11.56 ± 0.71	
Albumin (g/dL)	4.17 ± 0.15		4.28 ± 0.36	
Dry Weight (kg)	75.86 ± 7.45	75.8 ± 7.42	63.65 ± 13.13	62.64 ± 12.27
BMI (kg/m^2^)	25.54 ± 2.48	25.51 ± 2.67	22.80 ± 4.17	22.15 ± 3.97
WHR	0.97 + 0.05	0.97 ± 0.06	1.02 ± 0.43	1.00 ± 0.37
TBW	38.30 + 5.78	37.67 + 67	31.31 + 4.41	30.61 + 4.43
ECW	16.77 + 1.57	16.87 ± 1.96	14.07 + 2.12	13.54 ± 1.62
ICW	21.51 + 4.51	21.02 ± 3.30	17.22 + 2.67	16.61 ± 2.25
E/I	0.80 + 0.09	0.81 ± 0.10	0.82 + 0.10	0.83 ± 0.80
LTM (kg)	41.50 + 6.00	42.62 ± 6.66	38.72 + 4.89	38.57 ± 5.49
FAT (kg)	24.48 + 7.20	24.08 ± 6.68	22.95 + 10.73	22.31 ± 9.92

Note: All data are presented as mean ± SD. Statistical significance was set at *p* < 0.05. Abbreviations: CD, cold dialysis group SD standard dialysis group; Hct, Haematocrit; Hb, Haemoglobin (pre conditions measurements) BMI, Body mass index; WHR, waist hip-ratio; TBW, total body water; ECW, extracellular water, ICW, intracellular water; E/I, extracellular- intracellular water ratio; LTM, lean total mass. All variables showed normal distribution (*p* > 0.05). Within-group comparisons refer to pre- versus post-intervention changes within each group, while Δ-change analyses reflect between-group comparisons. The two groups were comparable at baseline, with no statistically significant differences observed for any of the assessed variables (all *p* > 0.05), although numerical differences in age and metabolic variables should be acknowledged given the small sample size.

**Table 2:** Insulin sensitivity indices according to study allocation.

Variables	CD	SD
OGIS (mL∙min^−1^∙m^−2^)		
Baseline	504.29 ± 90.47	622.43 ± 113.12
7-m post	588.86 ± 191.59	476.14 ± 103.72
Δ-Change (%)	16.77% ± 111.77	−23.50% ± 8.31 ^
*p* value	0.38	0.00
Effect size, d	0.49	1.16
AUCglucose disposal (0–120) (mg∙dL^−1^∙min^−1^)		
Baseline	160.59 ± 31.25	137.48 ± 36.54
7-m post	103.43 ± 15.81	124.84 ± 26.58
Δ-Change (%)	−35.59% ± 49.40 ^^	−9.19% ± 17.93
*p* value	0.00	0.30
Effect size, d	1.99	0.34
AUCinsulin disposal (0–120) (mg∙dL^−1^∙min^−1^)		
Baseline	59.06 ± 17.73	39.46 ± 33.03
7-m post	21.03 ± 8.38	41.39 ± 17.25
Δ-Change (%)	−64.34% ± 54.47 ^^^	4.90% ± 47.28
*p* value	0.00	0.86
Effect size, d	2.42	0.07

Note: Results were considered statistically significant when *p* < 0.05. Abbreviations: CD, cold dialysis and exercise group; SD, standard dialysis and exercise group. ^ Δ-Change CD vs. SD in OGIS: [F(1,11) = 9.019, *p* = 0.012]; ^^ Rate of Glucose disposal: [F(1,11) = 4.830, *p* = 0.050], ^^^ Rate of Insulin disposal: [F(1,11) = 4.824, *p* = 0.050]; Within-group comparisons refer to pre- versus post-intervention changes within each group, while Δ-change analyses reflect between-group comparisons.

**Table 3:** Post-Dialysis Blood pressure, nitrogen dioxide, and pre-dialysis heart rate data according to study allocation.

Variables	CD	SD
Post-Dialysis Systolic Pressure (mmHg)		
Baseline	127.29 ± 21.38	121.71 ± 22.45
7 m post	137.29 ± 20.14	126.71 ± 18.17
Δ-Change (%)	7.85% ± 5.79	4.10% ± 19.05
*p* value	0.05	0.30
Effect size, d	0.45	0.23
Post-Dialysis Diastolic Pressure (mmHg)		
Baseline	73.43 ± 12.82	69.29 ± 10.31
7 m post	75.29 ± 12.72	66.43 ± 11.96
Δ-Change (%)	2.53% ± 0.71	−4.12% ± 16.07
*p* value	0.72	0.59
Effect size, d	0.14	0.18
Post-Dialysis NO_2_ (ppb)		
Baseline	8.71 ± 2.62	5.86 ± 6.23
7 m post	6.43 ± 4.15	5.00 ± 4.96
Δ-Change (%)	−26.22% ± 58.22	−14.63% ± 20.27
*p* value	0.28	0.78
Effect size, d	0.61	0.14
Pre-Dialysis Heart Rate (BPM)		
Baseline	68.86 ± 18.21	79.00 ± 13.67
7 m post	73.00 ± 10.95	71.71 ± 9.14
Δ-Change (%)	6.01% ± 39.86	−9.22% ± 33.14
*p* value	0.38	0.13
Effect size, d	0.26	0.58

Note: Statistical significance was set at *p* < 0.05. Abbreviations: CD, cold dialysis and exercise group; SD, standard dialysis and exercise group, ppb, parts per billion; BPM, beats per minute. Within-group comparisons refer to pre- versus post-intervention changes within each group, while Δ-change analyses reflect between-group comparisons.

**Table 4:** Functional capacity according to study allocation.

Variables	CD (95% CI)	SD (95% CI)
STS-5 (min)		
Baseline	13.23 ± 7.16	12.20 ± 7.45
7 m post	12.45 ± 7.22	10.66 ± 7.81
Δ-Change (%)	−5.86 ± 0.78	−12.58
*p* value	0.35	0.22
Effect size, d	0.10	0.19
STS-60 (rep)		
Baseline	23.43 ± 8.50	26.43 ± 5.94
7 m post	27.43 ± 9.67	31.00 ± 8.26
Δ-Change (%)	17.07 ± 13.80	17.29 ± 39.16
*p* value	0.05	0.02
Effect size, d	0.41	0.59
6 m Fast Walk (sec)		
Baseline	4.45 ± 0.91	3.63 ± 1.58
7 m post	3.96 ± 0.77	3.36 ± 1.16
Δ-Change (%)	−10.99 ± −14.60	−7.31 ± −26.46
*p* value	0.18	0.45
Effect size, d	0.54	0.18
6 min Walk Test (m)		
Baseline	376.86 ± 112.59	389.14 ± 124.52
7 m post	416.57 ± 104.44	424.14 ± 117.70
Δ-Change (%)	10.53 ± −7.24	8.99 ± −5.46
*p* value	0.01	0.02
Effect size	0.34	0.27
Hand Grip (kg)		
Baseline	29.52 ± 6.76	27.15 ± 7.85
7 m post	31.29 ± 7.80	28.48 ± 9.64
Δ-Change (%)	5.97 ± 15.34	4.89 ± 22.81
*p* value	0.08	0.18
Effect size, d	0.23	0.14
Max test (watt)		
Baseline	35.71 ± 9.75	32.86 ± 14.96
7 m post	44.29 ± 15.11	42.86 ± 24.30
Δ-Change (%)	24 ± 54.91	30.43 ± 62.42
*p* value	0.08	0.04
Effect size, d	0.63	0.46

Note: STS-5: five sit to stand cycles; STS-60: sit to stand cycles in 60 s; Statistical significance was set at *p* < 0.05. Abbreviations: CD, cold dialysis and exercise group; SD, standard dialysis and exercise group. Within-group comparisons refer to pre- versus post-intervention changes within each group, while Δ-change analyses reflect between-group comparisons.

**Table 5:** Fatigue and Quality of life according to study allocation.

Variables	CD				SD			
	Before	After	*p* Values	Effect Size-d (Δ-Change%)	Before	After	*p* Values	Effect Size-d (Δ-Change%)
N	7	7	-	-	7	7	-	-
**SF-36 Quality of Life**								
Physical Function	75.71 ± 29.08	84.29 ± 22.11	0.13	0.29 (11.3% ± 25.5)	89.29 ± 18.41	92.14 ± 15.32	0.10	0.14 (3.19% ± 16.86)
Role Function	90.00 ± 10.35	95.00 ± 8.86	0.21	0.45 (5.5% ± 9.6)	96.43 ± 8.75	98.57 ± 3.50	0.35	0.28 (2.2% ± 6.12)
Body Pain	94.29 ± 7.28	93.33 ± 9.43	1.00	0.00 (−1% ± 8.3)	94.86 ± 8.20	97.71 ± 5.60	0.35	0.35 (3% ± 6.91)
General Health	71.57 ± 12.13	76.71 ± 16.27	0.24	0.31 (7.18% ± 14.2)	66.14 ± 14.53	80.29 ± 13.38	0.01	0.87 (21.3% ± 13.95)
Vitality	92.86 ± 13.59	95.71 ± 8.63	0.23	0.22 (3% ± 11.1)	78.57 ± 16.41	96.43 ± 5.15	0.03	1.26 (22.7% ± 10.78)
Role Emotional	93.14 ± 11.86	95.71 ± 6.78	0.35	0.23 (2.7% ± 9.32)	90.57 ± 14.91	93.14 ± 11.86	0.35	0.16 (2.8% ± 13.38)
Mental Health	77.71 ± 5.60	80.00 ± 4.66	0.40	0.38 (2.9% ± 5.13)	73.14 ± 5.11	78.71 ± 3.81	0.05	1.06 (7.6% ± 4.46)
Physical Health	81.43 ± 10.38	86.00 ± 11.46	0.11	0.36 (5.6% ± 10.92)	84.00 ± 10.43	92.14 ± 6.38	0.00	0.81 (9.6% ± 8.40)
Social Functioning	85.29 ± 3.53	87.29 ± 3.41	0.22	0.50 (2.3% ± 3.4)	81.43 ± 6.21	88.57 ± 4.92	0.00	1.10 (8.7% ± 5.56) *
SF-36 (Total)	87.57 ± 6.09	93.00 ± 3.21	0.00	0.96 (6.2% ± 4.65)	86.14 ± 7.40	92.43 ± 5.45	0.00	0.83 (7.3% ± 6.42)
Fatigue Severity Scale								
FSS	3.09 ± 1.06	2.97 ± 0.90	0.30	0.10 (−3.8% ± 0.98)	3.69 ± 0.99	3.61 ± 0.93	0.42	0.07 (−2.1% ± 0.96)

Note: Statistical significance was set at *p* < 0.05. Abbreviations: CD, cold dialysis and exercise group; SD, standard dialysis and exercise group. * CD vs. SD in Social Functioning: [F(1,14) = 5.834, *p* = 0.033]. Within-group comparisons refer to pre- versus post-intervention changes within each group, while Δ-change analyses reflect between-group comparisons.

## Data Availability

The original contributions presented in this study are included in the article. Further inquiries can be directed to the corresponding author.
